# A Hybrid Metadetector for Measuring Bell States of Optical Angular Momentum Entanglement

**DOI:** 10.3390/s24154817

**Published:** 2024-07-25

**Authors:** Yang Ming

**Affiliations:** 1 School of Electronic and Information Engineering, Changshu Institute of Technology, Suzhou 215000, China; njumingyang@gmail.com; 2 Department of Electrical and Computer Engineering, National University of Singapore, Singapore 117583, Singapore

**Keywords:** on-chip photonic metadetectors, optical angular momentum entanglement, transition metal dichalcogenide

## Abstract

High-dimensional entanglement of optical angular momentum has shown its enormous potential for increasing robustness and data capacity in quantum communication and information multiplexing, thus offering promising perspectives for quantum information science. To make better use of optical angular momentum entangled states, it is necessary to develop a reliable platform for measuring and analyzing them. Here, we propose a hybrid metadetector of monolayer transition metal dichalcogenide (TMD) integrated with spin Hall nanoantenna arrays for identifying Bell states of optical angular momentum. The corresponding states are converted into path-entangled states of propagative polaritonic modes for detection. Several Bell states in different forms are shown to be identified effectively. TMDs have emerged as an attractive platform for the next generation of on-chip optoelectronic devices. Our work may open up a new horizon for devising integrated quantum circuits based on these two-dimensional van der Waals materials.

## 1. Introduction

Quantum circuits are widely regarded as the most promising candidate for the next generation of information-processing platforms. Entanglement is an essential resource for producing quantum information applications in corresponding systems. In reality, entangled photon states have been extensively utilized for the demonstration of diverse quantum tasks. Various types of entangled states have been proposed based on different degrees of freedom of photons, including polarization [1,2], frequency [3], and angular momentum [4,5,6]. For the last one, Ref. [7] presents a comprehensive review. It is worth mentioning that photonic entanglement of optical angular momentum attracts considerable interest recently because the accordingly constructed high-dimensional entangled states have an enormous potential for enhancing the security and data capacity of quantum information systems [8,9]. The angular momentum of a photon can be divided into spin angular momentum (SAM) and orbital angular momentum (OAM) [10,11,12]; hence, there are SAM-OAM hybrid entanglement and OAM entanglement [6,13]. In order to make better use of entangled photons carrying angular momentum in practice, it is necessary to develop a compact platform for analyzing and detecting those states.

A reliable material base is crucial for designing on-chip detectors of optical angular momentum entanglement. Recently, monolayer transition metal dichalcogenides (TMDs) have emerged as an attractive candidate for novel integrated photonic and optoelectronic devices [14,15,16,17,18] due to their optically allowed direct bandgap at visible and near-infrared frequencies benefiting from atomic thin thickness [19]. In this article, we propose a hybrid metadetector of TMD monolayer integrated with spin Hall nanoantenna arrays for measuring maximally entangled states of optical angular momentum. TMD monolayers support robust excitons, which can interact with photons to form polaritonic modes known as exciton polaritons [20,21,22]. The light–matter interaction in the region of spin Hall nanoantenna arrays gives rise to chirality-dependent separation of photons, and different incident OAM states are coupled into their respective paths. Consequently, optical angular momentum entanglement is transformed into path entanglement carried by the guiding polaritonic modes. Through applicable geometry design, the propagation length of polaritonic modes is able to reach several tens of micrometers, which is long enough to ensure a fine performance of the metadetector. The influence of intrinsic loss is considered not to hinder the sorting of optical angular momentum entangled states. Several states in different forms are shown to be identified effectively. Our work goes a step further towards developing quantum applications of two-dimensional TMD materials and may open up a new horizon for devising quantum polaritonic devices.

## 2. Hybrid Metadetector Design

We have established that the total angular momentum of a photon includes SAM and OAM. A circularly polarized photon carries a SAM of *σħ*, where *σ* = ±1 for right- and left-handed circular polarization (RCP/LCP), respectively [11]. The quantity of OAM associated with helical-phase *exp*(*ilφ*) is *l*ℏ for each photon, where *l* is the quantum number of OAM defined as topological charge [11,23]. Different types of SAM-OAM hybrid entanglement and OAM entanglement have been demonstrated [6,13,24]. Here, we mainly focus on maximally entangled states, which represent an essential ingredient in most quantum information applications [25]. A bipartite entanglement of the following Bell state forms is first considered:

(1) $$\{\begin{array}{c} {|\Psi \rangle }_{hybrid}=\left(|R\rangle |l\rangle +|L\rangle |-l\rangle \right)/\sqrt{2}\\ {|\Psi \rangle }_{OAM}=\left(|l_{1}\rangle |l_{2}\rangle +|l_{2}\rangle |l_{1}\rangle \right)/\sqrt{2} \end{array}.$$

In these two equations, ${|\Psi \rangle }_{hybrid}$ and ${|\Psi \rangle }_{OAM}$ are Bell states of SAM-OAM hybrid entanglement and OAM entanglement, respectively.

**Figure 1:**
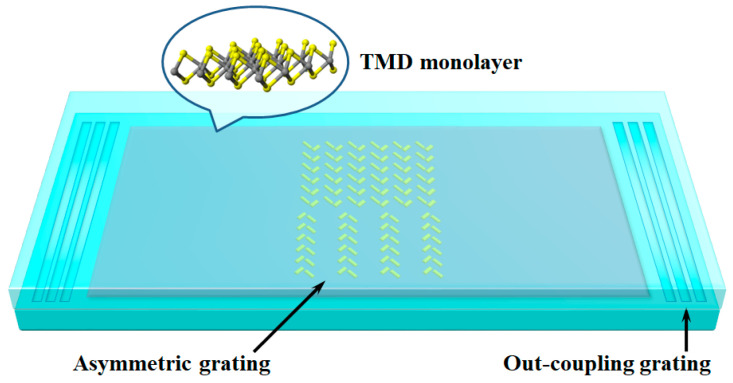
The schematic of the proposed hybrid metadetector consisting of a TMD monolayer integrated with asymmetric grating consisting of spin Hall nanoantenna arrays. This structure is sandwiched in the surrounding silica. Out-coupling gratings are utilized for signal collection. As an integrated platform, the overall size of the on-chip functional region is in the range of 10^1^~10^2^ μm. The TMD monolayer requires an effective area of a few tens of micrometers, while the spin Hall nanoantennas are on the subwavelength scale. In the thickness direction, the TMD monolayer is atomically thin, whereas the typical thickness of the nanoantennas is 10^1^~10^2^ nm. Compared to previous bulky systems for OAM entanglement analysis, this detector is especially compact.

Multiple efficient OAM sorting methods have been demonstrated in bulky systems [26,27,28]. For on-chip detection, several strategies have been proposed in the realm of classical optics [29,30,31,32]. The most effective method is using an asymmetric grating design [30], which can also be adopted to measure OAM entangled states. However, this method is inapplicable to the analysis of SAM; thus, additional design is necessary. In fact, TMD monolayers support spin-polarized valley transitions at the K and K’ points of the associated Brillouin zone owing to the broken inversion symmetry combined with time-reversal symmetry [33,34,35], which leads to the chiral coupling of spin photons. Nevertheless, as we need to combine the OAM degree of freedom, a more flexible scheme to deal with both SAM and OAM simultaneously is desired. Here, we choose to introduce spin Hall nanostructures [31,36] into the asymmetric grating for sorting SAM. Correspondingly, the schematic of a hybrid metadetector consisting of a TMD monolayer integrated with asymmetric grating consisting of spin Hall nanoantenna arrays is shown in Figure 1.

**Figure 2:**
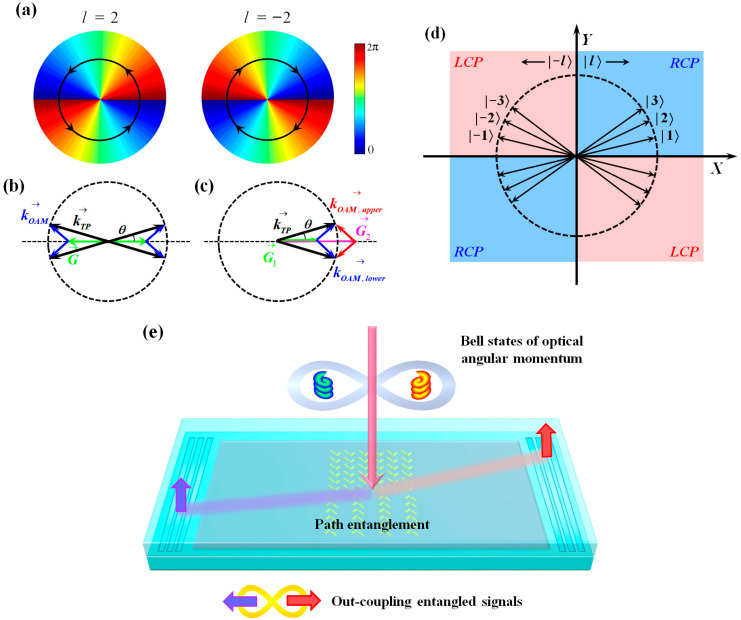
(**a**) The azimuthal *k*-vector carried by the OAM mode with *l* = 2 and *l* = −2. The spatial distribution exhibits circular symmetry. (**b**) The TMD polaritons excited by an OAM state launched on a single grating. The blue arrows depict the *k*-vector of the OAM modes. The single grating provides reciprocal vectors in two horizontal directions (green arrows), allowing the OAM modes to be coupled into four directions (black arrows) in this situation. (**c**) The excitation of TMD polaritons is limited to one side when an OAM state with positive topological charge is launched on an asymmetric grating. Since the upper and lower parts of the grating have distinct reciprocal vectors (green and magenta arrows), they are matched to azimuthal OAM *k*-vectors in different directions (blue and red arrows). (**d**) The general effect of decomposing the Bell states of SAM-OAM hybrid entanglement and OAM entanglement. (**e**) The functional illustration of the metadetector. The Bell states of SAM-OAM hybrid entanglement and OAM entanglement can be transferred into path entanglement for detection.

The asymmetric grating couples OAM states with different topological charges into their respective propagation directions on the chip [30,31]; thus, the OAM entanglement can be converted into path entanglement. To introduce the working principle, we should first clarify the azimuthal *k*-vector carried by the OAM mode [31], as is sketched in Figure 2a. The corresponding spatial distribution exhibits circular symmetry. When the OAM state is launched onto a grating, guiding polaritons can be excited through light–matter interaction when the *k*-vector matching condition is satisfied:

(2) $$\vec{k_{TP}}=\vec{G}+\vec{k_{OAM}}.$$

In this equation, ${\vec{k}}_{TP}$ denotes the wave vector of TMD polaritons, and $\vec{G}=2\pi /\Lambda$ is the reciprocal vector provided by the grating. ${\vec{k}}_{OAM}$ represents the *k*-vector of the incident OAM mode. In the situation of normal incidence, ${\vec{k}}_{OAM}$ can be expressed as [30,31]:

(3) $$\left|{\vec{k}}_{OAM}\right|=\frac{l\pi }{2r},$$

where *r* denotes the effective radius of the OAM mode field. Hence, the propagation direction of the excited propagative polaritonic mode can be defined according to the law of cosines, and the corresponding propagation angle *θ* is explicitly dependent on the topological charge *l*. As a result, OAM states with different absolute values of topological charge are coupled into distinct paths. However, the positive and negative signs of topological charge cannot be distinguished because the single grating provides reciprocal vectors in two horizontal directions, allowing propagative modes to be excited in four directions on both the left and right sides, as shown in Figure 2b. Therefore, an asymmetric grating design is introduced. The upper and lower parts have different periods, Λ_1_ and Λ_2_, respectively. They provide distinct reciprocal vectors; thus, they are matched to azimuthal OAM *k*-vectors in different directions. Therefore, the excitation of TMD polaritons by an OAM state with a certain positive or negative sign can be limited to one side of the asymmetric grating when the following condition is satisfied:

(4) $${\vec{k}}_{TP}={\vec{G}}_{1}+{\vec{k}}_{OAM,lower}={\vec{G}}_{2}+{\vec{k}}_{OAM,upper}.$$

The corresponding schematic is sketched in Figure 2c. To ensure the effective sorting of OAM, the center lines of the incident mode field and the asymmetric grating should be aligned. We can produce an OAM state with a positive topological charge by coupling it with TMD polaritons propagating to the right side. In contrast, when the OAM state has a negative topological charge, the generated TMD polaritons propagate towards the left.

To be able to further characterize SAM, we introduce spin Hall nanoantenna arrays as the built-in structures for the grating lines. They comprise gold nanoantenna pairs, whose orientations are ±*π*/4 and ∓*π*/4 in the upper and lower sections, respectively. The length, width, and height of a nanoantenna are 215 nm, 50 nm, and 40 nm. Each nanoantenna equivalent to a dipolar source brings a phase delay of Δ*φ*  =  ±*π*/4, and the distance between the central lines of the (+*π*/4)/(−*π*/4) subarrays is set to be *S* = 2/(*π*|*k_TP_*|); thus, the conditions for giving rise to chirality-dependent separation of spin photons are satisfied [36,37]. Overall, the general effect of decomposing the Bell states of SAM-OAM hybrid entanglement and OAM entanglement is shown in Figure 2d. The first, second, third, and fourth quadrants contain the LCP/positive (topological charge), RCP/negative, LCP/negative, and RCP/positive channels, respectively. Correspondingly, the functional illustration of the metadetector is exhibited in Figure 2e. The signals can be collected with the assistance of out-coupling gratings, and then the coincidence measurements are carried out to identify the forms of Bell states.

**Figure 3:**
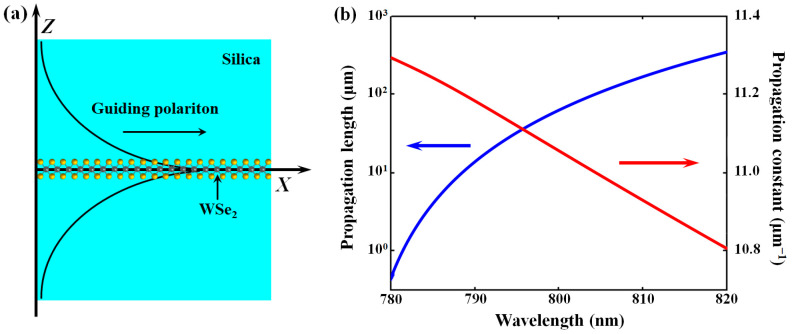
(**a**) The lateral geometry of the TMD waveguide section together with the guiding mode profile. (**b**) The curves of propagation constant *β* (red curve) and propagation length *L_p_* (blue curve) vary with wavelength.

The practical performance of the proposed metadetector depends on the geometry parameters, which need to be carefully chosen, especially the parameters of the planar TMD waveguide. We chose a tungsten diselenide (WSe_2_) monolayer for a detailed design, while the geometry parameters should also be correspondingly modulated if utilizing other TMDs such as tungsten disulfide (WS_2_) and molybdenum disulfide (MoS_2_). The TMD polaritonic modes can be classified into two families: TE modes and TM modes [38]. Here, we considered TE waveguide modes. The intrinsic loss of the modes can be reduced by enhancing the symmetry of the TMD waveguide; hence, the WSe_2_ monolayer is placed inside a silica surrounding, as is shown in Figure 3a. The corresponding mode field can be analyzed based on the waveguide electromagnetic theory. We used the following characteristic parameters of WSe_2_ for calculation: the resonance energy ℏ*ω*_0_ = 1.6 eV (A exciton) and the corresponding excitonic absorption *α_ω_0__* = 0.15 with an FWHM linewidth of ℏ*γ* = 50 meV [39]. The calculated TMD waveguide mode profile at 800 nm is sketched in Figure 3a. The curves of the propagation constant *β* and propagation length *L_p_*, varying with wavelength (780–820 nm), are plotted in Figure 3b. It can be seen that the propagation length is in the range of 10^1^–10^2^ μm, which is long enough for effective metadetector operation.

Comprehensively considering the common wavelength range of entangled photon sources, the intrinsic loss, and the effective mode width, the working wavelength was set at 800 nm. The corresponding propagation constant *β* is 11.07 μm^−1^; thus, Λ_1_ and Λ_2_ were chosen to be 515 nm and 632 nm. The distance between the edges of the asymmetric grating and the out-coupling gratings was chosen to be 60 μm based on three factors. This distance should not be too short to avoid the overlap of the adjacent signals for different OAM states [30,31], and it also should not be too long because of the intrinsic loss and the challenges of sample preparation. The period of out-coupling grating was set at 568 nm.

## 3. Entangled State Measurement

Down-converted photon pairs at 800 nm can be effectively provided by nonlinear optical crystals such as *β*-barium borate (BBO) [1,2], lithium niobate (LiNbO_3_) [40], and potassium titanyl phosphate (KTP) [41]. To further generate the Bell states of SAM-OAM hybrid entanglement and OAM entanglement, additional devices and elements, including a spatial light modulator (SLM), *q*-plate, and other wave plates are needed to modulate the wavefront and polarization of photon pairs. Several schemes have been demonstrated previously [4,13,42].

**Figure 4:**
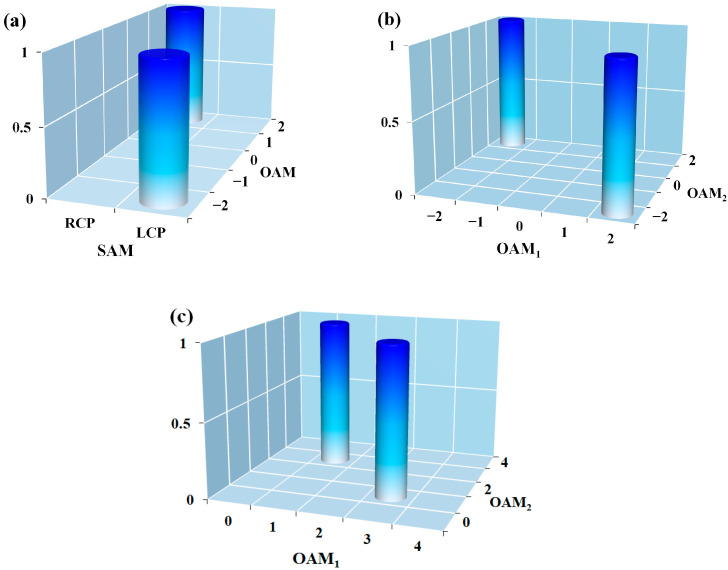
The coincidence histogram for the hybrid SAM-OAM Bell state (**a**) $\left(|R\rangle |2\rangle +|L\rangle |-2\rangle \right)/\sqrt{2}$, and OAM Bell states (**b**) $\left(|2\rangle |-2\rangle +|-2\rangle |2\rangle \right)/\sqrt{2}$, (**c**) $\left(|1\rangle |3\rangle +|3\rangle |1\rangle \right)/\sqrt{2}$.

The hybrid SAM-OAM Bell states can be prepared based on photon pairs, single photons, or even classical beams [13]. For classical beams such as cylindrical vector vortex beams (CVVBs) [31], nonseparability is established between the SAM and OAM degrees of freedom, which is treated as a classical analog of quantum entanglement. Our proposed metadetector can be employed for hybrid measurement of all these distinct types of Bell states. In practice, a genuine quantum background is usually preferred; hence, Bell states based on single photons are the most convenient and effective choice. In view of the generation strategies, the OAM topological charges of these states are usually ±*l*. Correspondingly, the path attenuation is symmetric when converting into guiding modes; hence, the influence of intrinsic loss for hybrid entanglement measurement does not have to be considered but the coincidence rate is drawn down by ~2 orders of magnitude. For this issue, ultra-bright photon sources can be chosen to ensure the detection efficiency. Here, we take $\left(|R\rangle |2\rangle +|L\rangle |-2\rangle \right)/\sqrt{2}$ as an example; the corresponding coincidence histogram is shown in Figure 4a.

For OAM entanglement, the situation is more complex, and the issue of SAM cannot be easily ignored. Although it is no longer a concerning degree of freedom, the SAM indeed influences the entanglement transfer. Therefore, we first set the same SAM for the entangled state, either RCP or LCP. Detailed considerations can be divided into two cases. If *l*_1_ + *l*_2_ = 0, the situation is still relatively simple to analyze. As |*l*_1_| = |*l*_2_|, the path attenuation is also symmetric; thus, the intrinsic loss does not influence the identification of states. As an illustration, the 3D histogram of $\left(|2\rangle |-2\rangle +|-2\rangle |2\rangle \right)/\sqrt{2}$ is shown in Figure 4b. The second case is *l*_1_ + *l*_2_ ≠ 0. It should be mentioned that |*l*_1_| = |*l*_2_| is not supposed to be chosen because entangled states degrade into product states with this condition. Correspondingly, we have |*l*_1_| ≠ |*l*_2_|, and the path attenuation becomes asymmetric. Therefore, the detection probabilities do not match in the two paths; thus, the coincidence results are expected to be affected. To deal with this problem, post-processing procedures are necessary. We choose a trigger gate processing. The gate shall be triggered when the most dissipative path with the largest absolute value of topological charge records an event. A signal shall be sent to the other path to open the detector for recording. After these procedures, the input state can still be successfully identified but the coincidence efficiency will be reduced by ~1 order of magnitude. Figure 4c exhibits the coincidence histogram of $\left(|1\rangle |3\rangle +|3\rangle |1\rangle \right)/\sqrt{2}$. In addition, it is worth mentioning that an upper limit of |*l*| can be expected for detection owing to the signal-to-noise ratio and the separation distance [31]. Due to the noise and dissipation effects during propagation, the output spot of each channel shall be broadened. The critical condition for OAM discrimination is *D*|tan(*θ_n_*_+1_) − tan(*θ_n_*)| > (*r_n_*_+1_ + *r_n_*)/2, where *D* is equal to the length of pure WSe_2_ waveguide region and *r_n_* is the radius of the output spot. This upper limit could increase through improving the device’s performance by placing nanowires at the corresponding channels [32], but it is more difficult to prepare the metadetector in this way.

We next consider a situation in which the polarization of the OAM entangled states is not RCP or LCP. It is common in practice that down-converted photons are linearly polarized. Under this circumstance, the entangled photons launched on the metadetector are coupled into bi-paths for the same OAM, namely RCP/*l* and LCP/*l*. It is not hard to ensure that the polarization of the incident states is horizontal or vertical. Correspondingly, the photon flux is equally distributed to both paths of RCP/*l* and LCP/*l* at a ratio of 50%. There are two ways to present the coincidence results. One is combining the coincidence counts from bi-paths, and the other is to distinguish the RCP/*l* and LCP/*l* channels. In fact, compared to the Bell state with asymmetric topological charges, which is mainly discussed here, the state $\left(|l_{1}\rangle |l_{1}\rangle +|l_{2}\rangle |l_{2}\rangle \right)/\sqrt{2}$ is more suitable for this scenario. Controlling the polarization of $\left(|l_{1}\rangle |l_{1}\rangle +|l_{2}\rangle |l_{2}\rangle \right)/\sqrt{2}$ shall make the coincidence measurement for it more flexible and convenient.

Overall, an effective integrated platform for detecting and analyzing Bell states of SAM-OAM hybrid entanglement and OAM entanglement is developed. Existing types of systems for this task have predominantly relied on bulky devices. In Ref. [6], Mair et al. used fork-shaped hologram gratings for OAM entanglement analysis. Although this method is effective, each fork grating could only sort OAM modes with specific topological charge. With the advent of spatial light modulators (SLMs), it has become more convenient to modulate holograms for OAM entanglement detection [7]. However, these devices are all essentially bulky, suffering from large volume and low resolution. In contrast, the compact metadetector proposed here could significantly advance the practical applications of OAM quantum systems.

## 4. Discussion and Conclusions

It is worth discussing the experimental feasibility of the proposed hybrid metadetector. There are three main functional structures: the TMD monolayer, the spin Hall nanoantenna arrays, and the out-coupling gratings. The latter two can be conveniently prepared at a high quality using current mature nanofabrication technologies, such as focused ion beam (FIB) and electron beam lithography (EBL) [43,44,45]. FIB etching technology is well-suited for carving out nano-scale hollow structures and can thus be employed to effectively machine nano-groove arrays for constructing the out-coupling gratings. For nano-block structures like the spin Hall nanoantenna arrays, EBL technology is currently one of the most powerful and widely used choices for fabrication. Moreover, another challenge is the processing technology for the WSe_2_ monolayer. For the preparation of high-quality, large-size TMD monolayer samples, the most commonly used approaches are exfoliation and synthesis [46,47]. Exfoliation is straightforward, requiring only manual shaking for a few seconds. However, the resulting products are quite random. In contrast, the synthesis approach using chemical vapor deposition is more efficient but involves more complex processes. Though the flexibility of device design is still limited to a certain extent, a series of intriguing functional optoelectronic devices with large-area TMD monolayers have been demonstrated [48]. Additionally, the functionality of the metadetector can also be further enhanced by incorporating other types of polaritons, such as plasmon polaritons [49,50] and phonon polaritons [51,52].

In conclusion, we propose a hybrid metadetector for detecting and analyzing optical angular momentum entangled states. The critical functional section of the metadetector is composed of a WSe_2_ monolayer integrated with spin Hall nanoantenna arrays. A silica surrounding is introduced to reduce the influence of intrinsic loss. The proposed device is used for the hybrid measurement of SAM-OAM hybrid Bell states and the detection of OAM Bell states. Several states in different forms are considered in detail and are effectively identified. Developing quantum applications of TMD monolayers has attracted a growing interest in recent years. Our work may open up a new horizon for designing quantum polaritonic devices based on two-dimensional van der Waals materials.

## Data Availability

The data presented in this study are available on request from the corresponding author.
